# Prostaglandin I_2_ is responsible for ameliorating prostaglandin E_2_ stress in stimulating the expression of tumor necrosis factor α in a β-amyloid protein -dependent mechanism

**DOI:** 10.18632/oncotarget.18462

**Published:** 2017-06-13

**Authors:** Shao-Qin Zheng, Zi-Yi Gong, Chen-Di Lu, Pu Wang

**Affiliations:** ^1^ The College of Life and Health Sciences, Northeastern University, Shenyang, P. R. China

**Keywords:** prostaglandin I_2_, prostaglandin E_2_, tumour necrosis factor α, β-amyloid protein, p38, Gerotarget

## Abstract

Cyclooxygenase-2 (COX-2) has been found to be induced during the early stage of Alzheimer’s disease (AD). Using mouse-derived astrocyte and APP/PS1 transgenic (Tg) mice as model systems, we firstly elucidated the mechanisms underlying COX-2 metabolic production including prostaglandin (PG)E_2_- and PGI_2_-mediated tumor necrosis factor α (TNF-α) regulation. Specifically, PGE_2_ accumulation in astrocyte activated the p38 and JNK/c-Jun signaling pathways *via* phosphorylation, resulting in TNF-α expression. In contrast, the administration of PGI_2_ attenuated the effects of PGE_2_ in stimulating the production of TNF-α by inhibiting the activity of TNF-α promoter and the binding activity of AP1 on the promoter of TNF-α. Moreover, our data also showed that not only Aβ_1-42_ oligomers but also Aβ_1-42_ fibrils have the ability to involve in mediating the antagonistic effects of PGE_2_ and PGI_2_ on regulating the expression of TNF-α *via* a p38- and JNK/c-Jun-dependent, AP1-transactivating mechanism. Reciprocally, the production of TNF-α finally accelerated the deposition of β-amyloid protein (Aβ)_1-42_ in β-amyloid plaques (APs), which contribute to the cognitive decline of AD.

## INTRODUCTION

Cycooxygenase-2 (COX-2) and prostaglandins (PGs) were found to be upregulated at the early stage of Alzheimer’s disease (AD) two decades ago [1]. Unfortunately, the initial mechanistic investigations of COX-2 and PGs in AD were disrupted by the failure of clinical trials of COX-2-specific inhibitors [2]. However, the results of these clinical trials have been called into question due to the lack of the guidance of a mechanistic study. Generally, COX-2 exhibits multiple biological functions and is thought to regulate the pathogenesis of AD *via* its metabolic products, including PGE_2_, PGD_2_ [and its dehydration end product 15-deoxy-∆^12,14^-PGJ_2_ (15d-PGJ_2_)], PGI_2_, PGF_2_α and TXA_2_ [3]. Among these PGs, the roles of PGE_2_ and PGI_2_ in neuroinflammation have been a focus of study [4], because these PGs potentially contribute to the production of β-amyloid protein (Aβ) and the hyperphosphorylation of tau in the brain [5, 6]. As indicated, COX-2 has been suggested to have a potential role of in neuroinflammation [7]. In addition, neuroinflammation is involved in Aβ deposition and tau phosphorylation [8], which contribute to the progression of AD.

The investigation of PGE_2_ in AD was initially prompted by the elevated levels of PGE_2_ in the cerebrospinal fluid (CSF) of AD patients [9]. The involvement of PGE_2_ in AD was shown to involve stimulation of Aβ production through the EP4 receptor [10]. Although this observation was called into question by clinical studies that showed that long-term treatment of AD patients with celecoxib or rofecoxib did not reduce Aβ content in the pathology of the disease [11, 12], we could not rule out their roles in suppressing the production of PGD_2_ or 15d-PGJ_2_, which exhibit anti-inflammatory functions and alleviate the effects of PGE_2_ stress on inflammation [13, 14]. Aside from the roles of PGE_2_ in neuroinflammation, PGI_2_ signalling was shown to facilitate joint inflammation in a mouse model of collagen-induced arthritis, while the administration of a PGI_2_ antagonist reduced pain and inflammation in rodent models of hyperalgesia and chronic arthritis [15]. In contrast to the seemingly pro-inflammatory properties of PGI_2_, its effects in certain conditions are still debated [5]. For example, PGI_2_ has been studied as a potentially important suppressor of allergen-induced inflammation [5]. Thus, the effects of PGI_2_ on the inflammatory reactions remain uncertain in peripheral or central tissues.

Although we could not find direct evidence of the relationship between PGI_2_ and neuroinflammation, a growing body of research has revealed that PGI_2_ has the ability to regulate the synthesis of cytokines [16]. For example, treatment with PGI_2_ analogs, including iloprost and Treprostinil suppressed tumour necrosis factor α (TNF-α) expression in human myeloid dendritic cells [16]. In contrast, PGE_2_ treatment elevated the expression of TNF-α in SH-SY5Y cells [14]. However, the antagonistic regulatory mechanisms of TNF-α that are mediated by PGE_2_ and PGI_2_ during the course of AD development are not often studied. Although little is known about the relationship between PGs and TNF-α, TNF-α has been suggested to regulate the cleavage of amyloid precursor protein (APP) [17-20]. For example, TNF-α treatment upregulates the expression of BACE-1 in APPsw Tg mice [20]. Additionally, TNF-α stimulates γ-cleavage of APP in HEK293 cells [18]. For the reason, TNF-α is thought to have the ability to induce the expression or phosphorylation of γ-secretases, including presenilin (PS) 1, PS2 and nicastrin (NCT) in HEK293 or human neuronal cells [17, 19]. When considered together, these data prompted us to investigate the roles of PGE_2_ and PGI_2_ in regulating the expression of TNF-α during the course of AD development.

Accordingly, we demonstrated that PGE_2_ induction during the early stage of AD stimulated the expression of TNF-α *via* an Aβ_1-42_-dependent, AP1-activating pathway. In contrast, PGI_2_ attenuated the effects of PGE_2_ in stimulating the expression of TNF-α by depressing the activity of the p38 and JNK/c-Jun pathways. In addition, not only Aβ oligomers but also Aβ fibrils had the ability to stimulate the expression of TNF-α. Reciprocally, TNF-α accumulation in or secretion from astrocytes accelerated Aβ deposition in APs.

## RESULTS

### TNF-α is markedly upregulated in the brains of AD patients and APP/PS1 transgenic mice

**Figure 1:**
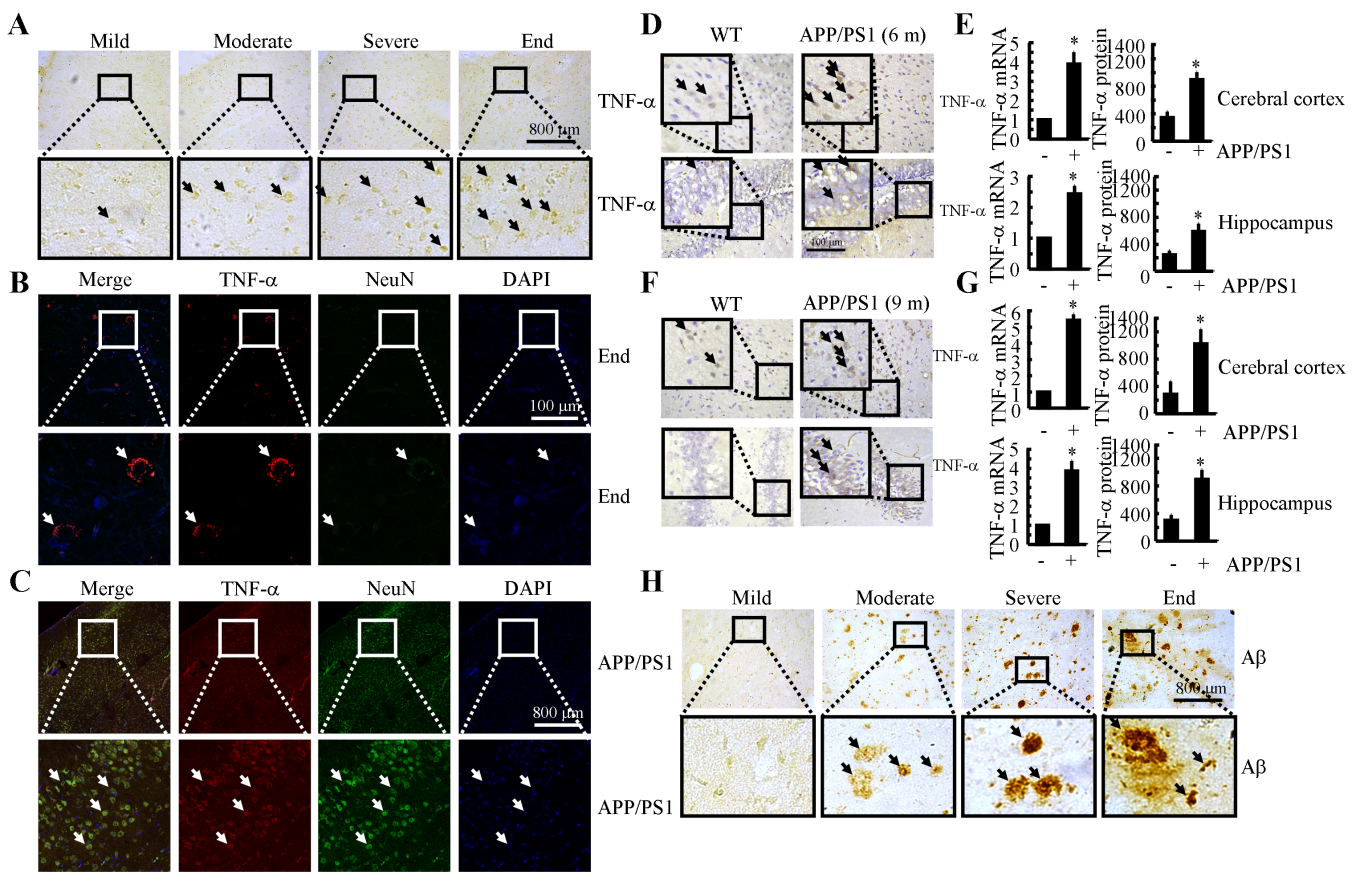
TNF-α expression was progressively elevated with Aβ deposition in APs during the course of AD development **A.**, **B.**, **H.** The tissue blocks of human brains at different stages of AD were collected by the New York Brain Bank at Columbia University. Free-floating slices (40 µm) were prepared by cryostat (*n* = 1). **A.**, **H.** The immunoreactivity of TNF-α and Aβ was determined by immunohistochemistry using an anti-TNF-α or -Aβ antibody. The arrows demonstrated the positive staining of TNF-α or Aβ. **B.** The slices of human or mouse brains were double-stained with TNF-α (red) or NeuN (green) antibodies before being observed under confocal microscopy. **C.**- **G.** The brains of WT or APP/PS1 transgenic mice at 6 or 9 months of age were collected following anesthesia and perfusion (*n* = 9). **C.** The slices of human or mouse brains were double-stained with TNF-α (red) or NeuN (green) antibodies before being observed under confocal microscopy. **D.**, **F.** The immunoreactivity of TNF-α was determined by immunohistochemistry using an anti-TNF-α antibody. The arrows demonstrated the positive staining of TNF-α. **E.**, **G.** TNF-α protein and mRNA levels were determined by TNF-α enzyme immunoassay kits and qRT-PCR, respectively. Total amounts of protein and GAPDH served as an internal control. *, *p < 0.05* with respect to WT control.

Because previous studies have suggested that TNF-α aggravates the pathogenesis of AD [21], we evaluated the expression levels of TNF-α in AD patients and APP/PS1 transgenic mice at 6 or 9 months of age. As shown in Figure 1A, TNF-α immunostaining progressively increased throughout the course of AD development. Interestingly, morphological analysis demonstrated that positive staining for TNF-α was located in the neurons (Figures. 1B, 1C). In line with these observations in AD patients, in 6-month-old APP/PS1 mice, TNF-α immunostaining was strongly increased in the cerebral cortex and dentate gyrus (DG) region of the hippocampus compared to that in WT C57BL/6 mice (Figure 1D). To further confirm this finding, we examined the mRNA and protein levels of TNF-α in these APP/PS1 Tg mice by qRT-PCR and ELISA. In agreement with the immunostaining data, our results demonstrated the upregulation of TNF-α mRNA and protein levels in the cerebral cortex and DG region of the hippocampus (Figure 1E). In addition, we found that TNF-α was also increased in the APP/PS1 mice at 9 months of age (Figure 1F). Similarly, the mRNA and protein levels of TNF-α were sustained above basal levels (Figure 1G). These data reveal that TNF-α is upregulated with the development/progression of AD. As Aβ is progressively deposited in β-amyloid plaques (APs) during the course of AD development (Figure 1H), our data indicate a possible role of Aβ aggregation in TNF-α stimulation.

### TNF-α was either synthesized in neurons or translocated from astrocytes during the course of AD development

**Figure 2:**
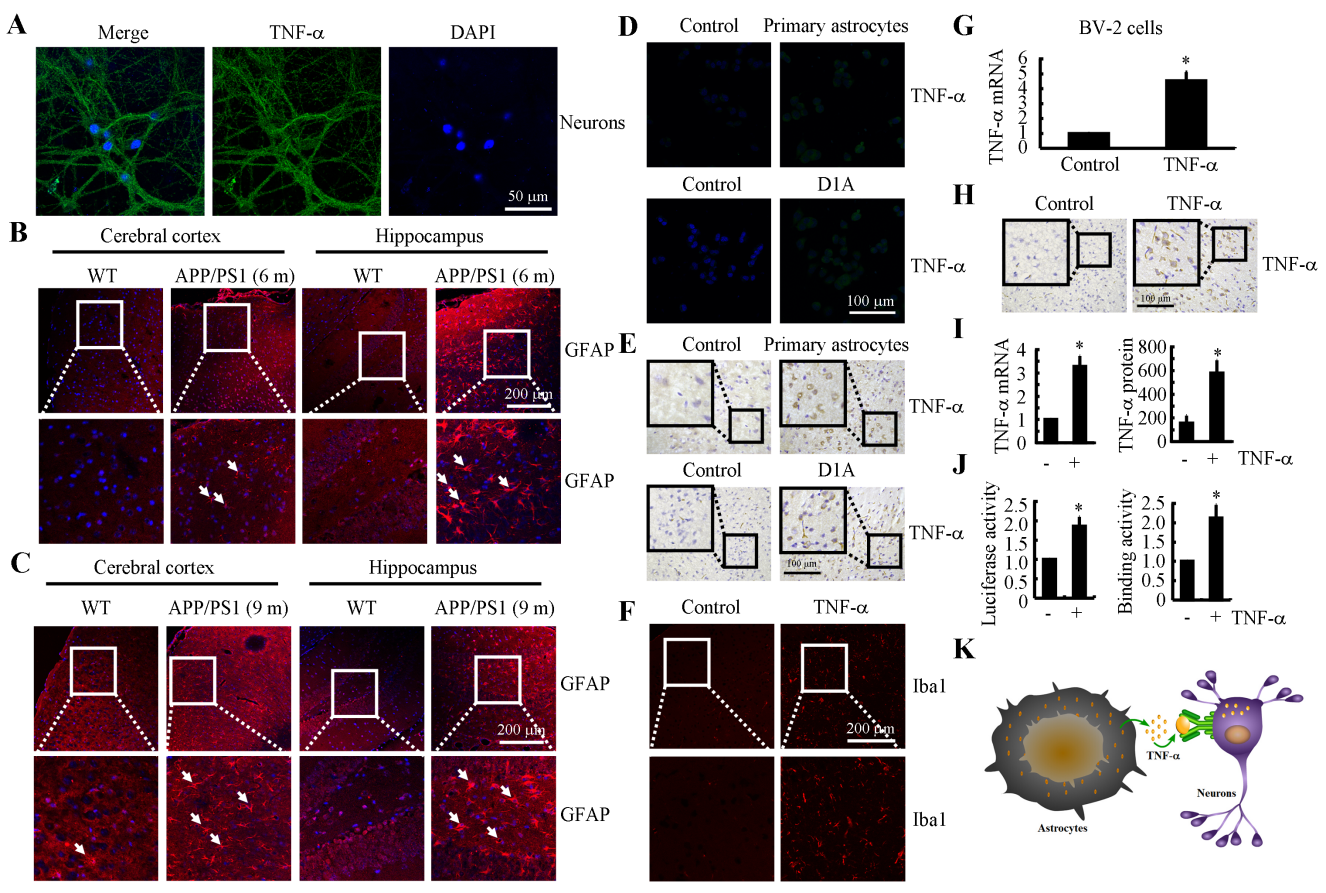
TNF-α was either synthesized in neurons or translocated from astrocytes during the course of AD development **A.** The neurons were separated and cultured from the hippocampus of new-born C57BL/6 mice. The cells were stained with TNF-α antibody and DAPI. **B.**, **C.**The brains of WT or APP/PS1 transgenic mice at 6 or 9 months of age were collected following anaesthetization and perfusion of the mice (*n* = 10). The activity of astrocytes was determined by staining with GFAP. **D.**, **E.** n2a cells or freshly collected slices were co-cultured with primary astrocytes or D1A cells. The immunoreactivity for TNF-α was determined *via* immunofluorescence or immunohistochemistry. **F.** TNF-α (10 ng/5 µl) was injected (i.c.v.) into the ventricles of WT mice before the brains were collected (*n* = 6). The activity of microglia cells was determined by immunofluorescence. **G.** BV-2 cells were incubated with TNF-α for 24 h. The expression of TNF-α was determined by qRT-PCR. **H.**, **I.** TNF-α (10 ng/5 µl) was injected (i.c.v.) into the ventricles of WT mice before the brains were collected (*n* = 6). TNF-α immunoreactivity was revealed with an anti-TNF-α antibody. TNF-α protein and mRNA levels were determined by TNF-α enzyme immunoassay kits and qRT-PCR, respectively. **J.** D1A cells were treated with TNF-α (10 ng/ml) for 24 h. (J left panels) The activity of the TNF-α promoter was determined using luciferase activity kits. (J right panels) The binding activity of AP-1 to the promoter of TNF-α was determined by ChIP assay. **K.** The model of TNF-α origination. *, *p < 0.05* with respect to the vehicle-treated control.

We next aimed to elucidate the origination of TNF-α during the progression of AD. As a result, the primary cultured neuronal cells showed positive TNF-α staining (Figure 2A). However, these observations could not exclude that high levels of TNF-α might be produced by astrocytes, which in turn translocate to neuronal cells. To verify this hypothesis, we next showed that the activity of astrocyte was stimulated in 6- and 9-month-old APP/PS1 Tg mice (Figures 2B, 2C). More specifically, we co-cultured primary cultured astrocytes or D1A cells in the upper chamber of transwells while culturing n2a cells in the lower chamber. After 24 h, TNF-α immunofluorescence was enhanced in the n2a cells (Figure 2D). To further confirm this observation, experiments were carried out to determine if TNF-α secreted from primary cultured astrocytes or D1A cells had the ability to bind to neurons of cultured slices of C57BL/6 mouse brains. As expected, our data revealed that TNF-α staining was elevated in neurons of the C57BL/6 brains that were co-cultured with astrocytes (Figure 2E). In addition, the highly induced TNF-α could stimulate the activity of microglia (Figure 2F), which further induced the expression of TNF-α in microglia (Figure 2G). Moreover, TNF-α injection (i.c.v.) was also found to induce the production of additional TNF-α in the cerebral cortex of WT mice (Figure 2H). To exclude the possibility that the increasement of TNF-α was caused by the binding of exogenous TNF-α on neurons, the mRNA and protein levels of TNF-α were further verified by qRT-PCR and ELISA (Figure 2I). In D1A cells, the luciferase activity and binding activity of AP-1 on the TNF-α promoter were determined, and the results demonstrated that the promoter and binding activity were elevated after TNF-α treatment (Figure 2J). Therefore, it is clear that TNF-α was either synthesized in neurons or translocated from astrocytes during the course of AD development (Figure 2K).

### COX-2 induces the expression of TNF-α by altering the balance between PGE_2_ and PGI_2_ in APP/PS1 Tg mice

**Figure 3:**
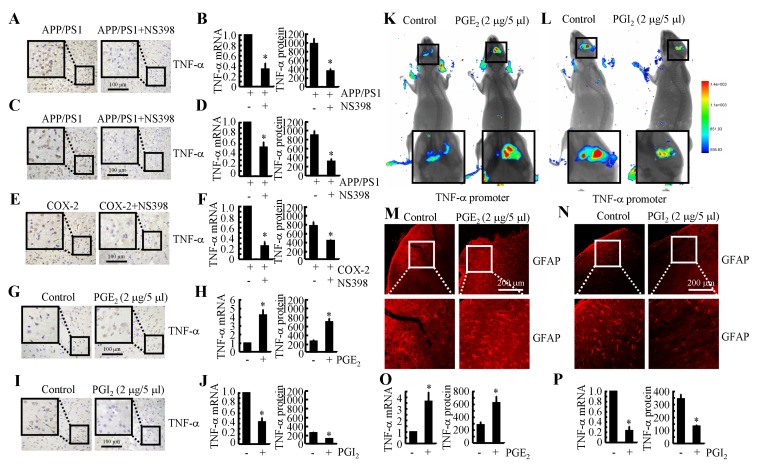
COX-2 mediated the opposing effects of PGE_2_ and PGI_2_ in regulating the expression of TNF-α in APP/PS1 Tg mice **A.**, **B.** APP/PS1 or (E, F) COX-2 transgenic mice at the age of 3 months received NS398 (50 µg/20 µl/d) for 6 months before the brains were harvested (*n* = 6). **C.**, **D.** The APP/PS1 mice were intracerebroventricularly (i.c.v.) injected with NS398 (2 µg/5 µl) for 24 h (*n* = 6). In select experiments, the WT or APP/PS1 mice were injected (i.c.v.) with PGE_2_ **G.**, **H.** or PGI_2_ **I.**, **J.**, respectively, for 24 h (*n* = 6). **A.**, **C.**, **E.**, **G.**, **I.** TNF-α immunoreactivity was determined *via* immunohistochemistry with an anti-TNF-α antibody. **B.**, **D.**, **F.**, **H.**,**J.**-**L.** TNF-α protein and mRNA levels were determined using TNF-α enzyme immunoassay kits and qRT-PCR, respectively. The total amount of protein and GAPDH served as internal controls. **K.**, **L.** the cerebral ventricle in one hemisphere was injected with PGE_2_ (2 µg/5 µl) or PGI_2_ (2 µg/5 µl), and the cerebral ventricle on the other side was injected (i.c.v.) with D1A cells that had been pre-transfected with the TNF-α promoter (*n* = 4). Luciferase activity was measured in the different groups of mice using a live animal imaging system. **M.**, **N.** the WT or APP/PS1 mice were injected (i.c.v.) with PGE_2_ or PGI_2_, respectively, for 24 h (*n* = 6). GFAP immunofluorescence was revealed with an GFAP-specific antibody and observed under a microscope. **O.**, **P.** D1A cells were treated with PGE_2_ or PGI_2_ for 24 h before total RNA was extracted. TNF-α enzyme immunoassay kits and qRT-PCR, respectively. The total amount of protein and GAPDH served as internal controls. *, *p < 0.05* with respect to the vehicle-treated control.

Because COX-2 expression is elevated during the early stage of AD and is associated with Aβ deposition [22], we studied whether COX-2 inhibition by NS398 could downregulate the expression of TNF-α. We intranasally administered NS398 (50 µg/kg/d) to APP/PS1 mice for 6 months before they were sacrificed. The results demonstrated that NS398 administration decreased the mRNA and protein expression of TNF-α (Figures 3A, 3B). To further validate the above results, we injected (i.c.v.) APP/PS1 mice at 6 months of age with NS398 (2 µg/5 µl). After 24 h, the brains of the mice were collected, and the expression of TNF-α was determined. The mRNA and protein expression of TNF-α was highly induced in the APP/PS1 mice, and this effect was blocked by NS398 injection (Figures 3C, 3D). These observations clearly indicate that COX-2 elevation in APP/PS1 Tg mice stimulated the expression of TNF-α.

To furhter validate the critical roles of COX-2 in mediating the effects of APP/PS1 overexpression on upregulating the expression of TNF-α, experiments were carried out in COX-2 Tg mice. The results demonstrated that COX-2 overexpression induced the expression of TNF-α in C57BL/6 mice (Figures 3E, 3F). The strong induction of TNF-α expression was markedly attenuated by NS398 treatment in COX-2 Tg mice (Figures 3E, 3F). This observation not only confirmed the critical role of COX-2 in the upregulation of the expression of TNF-α in the APP/PS1 and COX-2 Tg mice but also indicated the possible effects of COX-2 metabolic products, including PGE_2_ and PGI_2_, in regulating the expression of TNF-α. In addition, it was evident that PGE_2_ (2 µg/5 µl) injection (i.c.v.) stimulated the expression of TNF-α in the cerebral cortex of the mice (Figures 3G, Supplementary Figure 1A). The mRNA and protein levels of TNF-α were detected using qRT-PCR and ELISA. The results showed that PGE_2_ injection (i.c.v.) increased the expression of TNF-α in the cerebral cortex of the WT mice (Figure 3H). To further verify the key role of PGE_2_ in upregulating the expression of TNF-α *in vivo*, we combined i.c.v. injection with live animal imaging. As described in Figure 3K, D1A cells that were transfected with TNF-α promoter constructs were pre-seeded in the right lateral ventricle of WT mice at 6 months of age, whereas PGE_2_ (2 µg/5 µl) was injected into the left ventricle of the same mice. After 24 h, luciferin was injected (i.c.v.) into the same ventricle, and live animal imaging was then conducted. The results showed that PGE_2_ increased the luciferase activity of the TNF-α promoter (Figure 3K). To understand if the increased production of TNF-α resulted from astrocyte activation, we assessed the activity of astrocytes following i.c.v. injection of PGE_2_. The results demonstrated that astrocytes were markedly stimulated by PGE_2_ injection (Figure 3M).

To further understand the role of COX-2 metabolic products in TNF-α regulation, we similarly injected (i.c.v.) PGI_2_ into the ventricles of 6-month-old APP/PS1 mice. In contrast to PGE_2_ injection, PGI_2_ injection (i.c.v.) decreased the positive staining of TNF-α in the cerebral cortex of the APP/PS1 transgenic mice at 6 months of age (Figure 3I). mRNA and protein levels of TNF-α were assessed using qRT-PCR and western blotting. Results similar to those of the IHC assays were obtained (Figures 3J, Supplementary Figure 1B). Additionally, PGI_2_ treatment actively altered the transcriptional activity of the TNF-α promoter and the synthesis of TNF-α in live animals, as observed by live animal imaging (Figure 3L). We then sought to understand the role of PGI_2_ in regulating the expression of TNF-α through the activity of astrocyte by quantifying astrocytel activity following the injecting (i.c.v.) of PGI_2_. As expected, the activity of astrocyte cells was suppressed by PGI_2_ injection (i.c.v.) (Figure 3N). These observations not only demonstrated the opposing roles of PGE_2_ and PGI_2_ in regulating the expression of TNF-α but also indicated the possible roles of astrocyte in the expression of TNF-α. To further confirm the important roles of astrocyte in the production of TNF-α, PGE_2_ and PGI_2_ were used to treat D1A cells, and the results demonstrated that PGE_2_ treatment increased the expression of TNF-α and that PGI_2_ treatment decreased the expression of TNF-α in D1A cells (Figures 3O, 3P). Thus, it is clear that COX-2 overexpression in APP/PS1 Tg mice upregulates the expression of TNF-α *via* the antagonistic effects of PGE_2_ and PGI_2_ on the activity of astrocyte.

### The p38 and JNK/c-Jun signalling pathways are critical for mediating the effects of PGE_2_ and PGI_2_ in regulating the expression of TNF-α in astrocyte

**Figure 4:**
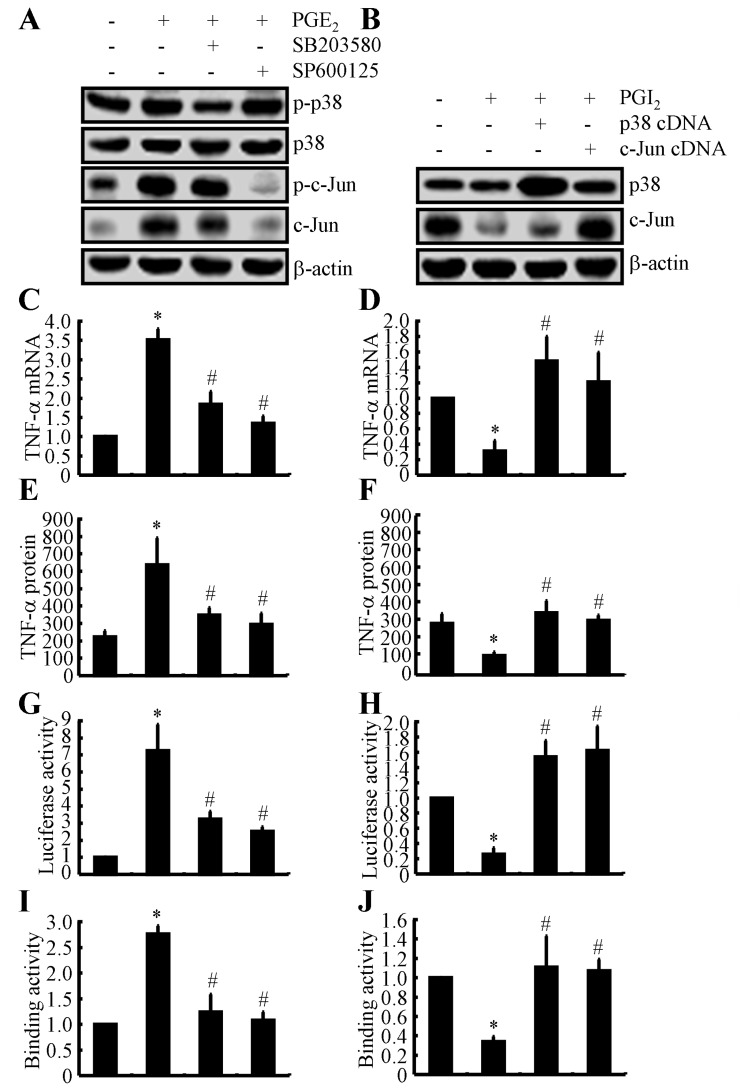
Critical role of the p38 and JNK/c-Jun pathways in regulating the expression of TNF-α in PGE_2_- and PGI_2_-treated D1A cells **A.**, **C.**, **E.**, **G.**, **I.** Mouse D1A astrocyte were treated with PGE_2_ (10 µM) in the absence or presence of the p38 inhibitor SB203580 (10 µM) or the JNK inhibitor SP600125 (10 µM) for 24 h before protein or mRNA was extracted. **A.** Levels of phosphorylated p38, total p38, phosphorylated c-Jun and total c-Jun were detected by immunoblotting using specific antibodies. Equal lane loading was demonstrated by the similarity in the intensity of the band for total β-actin. **C.**, **E.** TNF-α mRNA and protein levels were determined using TNF-α enzyme immunoassay kits and qRT-PCR, respectively. **G.** The activity of the TNF-α promoter was determined using a luciferase activity kit. **I.** The binding activity of AP-1 to the promoter of TNF-α was determined by ChIP assay. **B.**, **D.**, **F.**, **H.**, **J.** The D1A cells were pre-transfected with p38 or c-Jun cDNA before being treated with PGI_2_ (10 µM) for 24 h. **B.** Levels of total p38 and c-Jun were detected by immunoblotting using specific antibodies. Equal lane loading was demonstrated by the similarity in the intensity of the band for total β-actin. **D.**, **F.** TNF-α mRNA and protein levels were determined using TNF-α enzyme immunoassay kits and qRT-PCR, respectively. **H.** The activity of the TNF-α promoter was determined using a luciferase activity kit. **J.** The binding activity of AP-1 to the promoter of TNF-α was determined by ChIP assay. The data represent the mean ± S.E. of three independent experiments. *, *p < 0.05* with respect to the vehicle-treated or vector-transfected control. #, *p < 0.05* compared to treatment with PGE_2_-or PGI_2_ alone.

Because PGE_2_ and PGI_2_ exhibited antagonistic effects on regulating the expression of TNF-α, we next determined the mechanism of TNF-α regulation by PGE_2_ and PGI_2_. Using D1A cell cultures, we found that PGE_2_ treatment induced the phosphorylation of p38 without altering the total protein levels of p38 in D1A cells (Figure 4A). To further elucidate the potential role of p38 in regulating the expression of TNF-α, we treated D1A cells with the pharmacological p38 inhibitor SB203580 (10 µM) in the absence or presence of PGE_2_ (10 µM). Incubation of D1A cells with SB203580 (10 µM) not only suppressed the PGE_2_-induced phosphorylation of p38 but also reversed the PGE_2_-dependent decrease in TNF-α synthesis (Figures 4C, 4E). Similar results were obtained when we treated D1A cells with the JNK inhibitor SP600125 (10 µM) in the presence of PGE_2_ (10 µM) (Figures 4A, 4C, 4E). In contrast to PGE_2_ treatment, PGI_2_ (10 µM) treatment decreased the phosphorylation of p38 and c-Jun, which resulted in TNF-α suppression in D1A cells (Figure 4B). When we overexpressed p38 and c-Jun in D1A cells, the levels of TNF-α were restored to basal levels (Figures 4B, 4D, 4F).

To identify the mechanism of the transcriptional upregulation of TNF-α by PGE_2_ and PGI_2_, we investigated the possible involvement of transcription factors in this process. In previous studies [23], AP1 was shown to be involved in regulating the synthesis of TNF-α. Consistent with these previous studies, we found that PGE_2_ and PGI_2_ regulated the luciferase activity of the TNF-α promoter in opposite directions (Figures 4G, 4H). In addition, these data were further confirmed using chromatin immunoprecipitation assays (Figures 4I, 4J). Therefore, these data provide concrete support for the notion that PGE_2_ and PGI_2_ have antagonistic effects on the regulation of TNF-α expression *via* a p38- and JNK/c-Jun-dependent AP-1-activating mechanism.

### Aβ production is involved in mediating the antagonistic effects of PGE_2_ and PGI_2_ in regulating the expression of TNF-α in the cerebral cortex of APP/PS1 Tg mice

**Figure 5:**
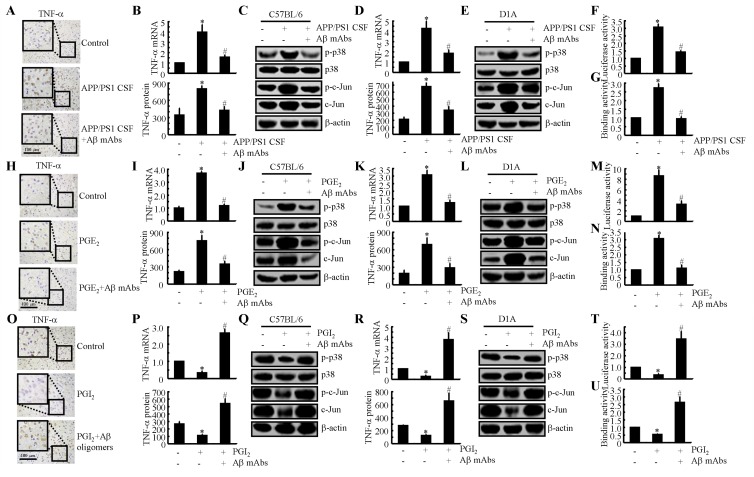
Aβ_1-42_ mediated the antagonistic effects of PGE_2_ and PGI_2_ in regulating the expression of TNF-α **A.**-**C.** Cerebrospinal fluid (CSF) was collected from APP/PS1 mice at 6 months of age and then injected (i.c.v.) into WT C57BL/6 mice in the absence or presence of Aβ antibodies (1 µg/5 µl). After two weeks, the mice were sacrificed (*n* = 4). **H.**-**J.** The WT mice were injected with PGE_2_ (2 µg/5 µl) in the absence or presence of Aβ antibodies (1 µg/5 µl) for 24 h (*n* = 6). **O.**-**Q.** The APP/PS1 mice were injected with PGI_2_ in the absence or presence of Aβ (1 µg/5 µl) for 24 h (*n* = 6). **D.**-**G.**, **K.**-**N.**, **R.**-**U.** Similar treatments were applied to D1A cells. (A, H, O) TNF-α immunoreactivity was determined *via* immunohistochemistry. **B.**, **I.**, **P.**, **D.**, **K.**, **R.** TNF-α protein and mRNA levels were determined by TNF-α enzyme immunoassay kits and qRT-PCR, respectively. **C.**, **J.**, **Q.**, **E.**, **L.**, **S.** Levels of phosphorylated p38, total p38, phosphorylated c-Jun and total c-Jun were detected by immunoblotting using specific antibodies. Equal lane loading was demonstrated by the similarity in the intensity of the band for total β-actin. **F.**, **M.**, **T.** The activity of the TNF-α promoter was determined by luciferase activity kits. **G.**, **N.**, **U.** The binding activity of AP-1 to the promoter of TNF-α was determined by ChIP assay. *, *p < 0.05* with respect to the vehicle-treated control. #, *p < 0.05* compared to treatment with PGE_2_ or PGI_2_ alone.

Due to the essential role of Aβ_1-42_ in neuroinflammation [5, 6], we sought to determine the involvement of Aβ_1-42_ in mediating the effects of PGE_2_ and PGI_2_ in regulating the expression of TNF-α. To further understand the role of Aβ_1-42_ in TNF-α regulation, we injected CSF from 6-month-old APP/PS1 mice age into WT mice in the absence or presence of an Aβ antibody (1 µg/5 µl). After two weeks, the mice were sacrificed, and the expression of TNF-α was determined. Our data revealed that APP/PS1 CSF injection (i.c.v.) elevated the expression of TNF-α and that this elevation was blocked by the Aβ antibody, as revealed by immunohistochemistry (Figure 5A). mRNA and protein levels of TNF-α were assessed using qRT-PCR and western blotting, and these assays revealed results similar to those of the IHC assays (Figure 5B). In addition, the CSF of the APP/PS1 Tg mice had the ability to stimulate the phoshorylation of p38 and c-Jun in the WT mice, and this effect was reversed by the addition of the Aβ mAbs (Figure 5C). Similar results were also obtained in APP/PS1 CSF-treated D1A cells (Figures 5D, 5E). Promoter and ChIP assays further verified that Aβ in the CSF of APP/PS1 mice has the ability to stimulate the transcriptional or translational activity of TNF-α in D1A cells (Figures 5F, 5G).

To further verify the above results, we injected (i.c.v.) PGE_2_ (2 µg/5 µl) into the ventricles of WT mice in the absence or presence of an Aβ_1-42_ antibody (1 µg/5 µl). The results demonstrated that the Aβ_1-42_ antibody thoroughly diminished the stimulatory effects of PGE_2_ on TNF-α expression (Figures 5H, 5I). In addition, the phosphorylation of p38 and c-Jun was attenuated by the injection of Aβ mAbs (1 µg/5 ml) for 24 h (Figure 5J). The observations were further confirmed in D1A cells (Figures 5K-5N). In contrast to PGE_2_ treatment, PGI_2_ treatment showed inhibitory effects on TNF-α expression in APP/PS1 Tg mice (Figures 5O, 5P). When we added Aβ_1-42_ oligomers to the ventricles of APP/PS1 Tg mice, the levels of TNF-α were restored to basal levels (Figures 5O, 5P). The p38 and c-Jun signalling pathways were once again involved in mediating TNF-α synthesis in the Tg mice (Figure 5Q). In line with these *in vivo* results, *in vitro* observations also supported this notion and confirmed the role of AP1 in mediating TNF-α synthesis (Figures 5R-5U). Therefore, the production of Aβ_1-42_ involved in the roles of PGE_2_ and PGI_2_ in regulating the expression of TNF-α in APP/PS1 Tg mice, an experimental AD model.

### Not only Aβ_1-42_ oligomers but also Aβ_1-42_ fibrils stimulate the expression of TNF-α in the brains of APP/PS1 mice

**Figure 6:**
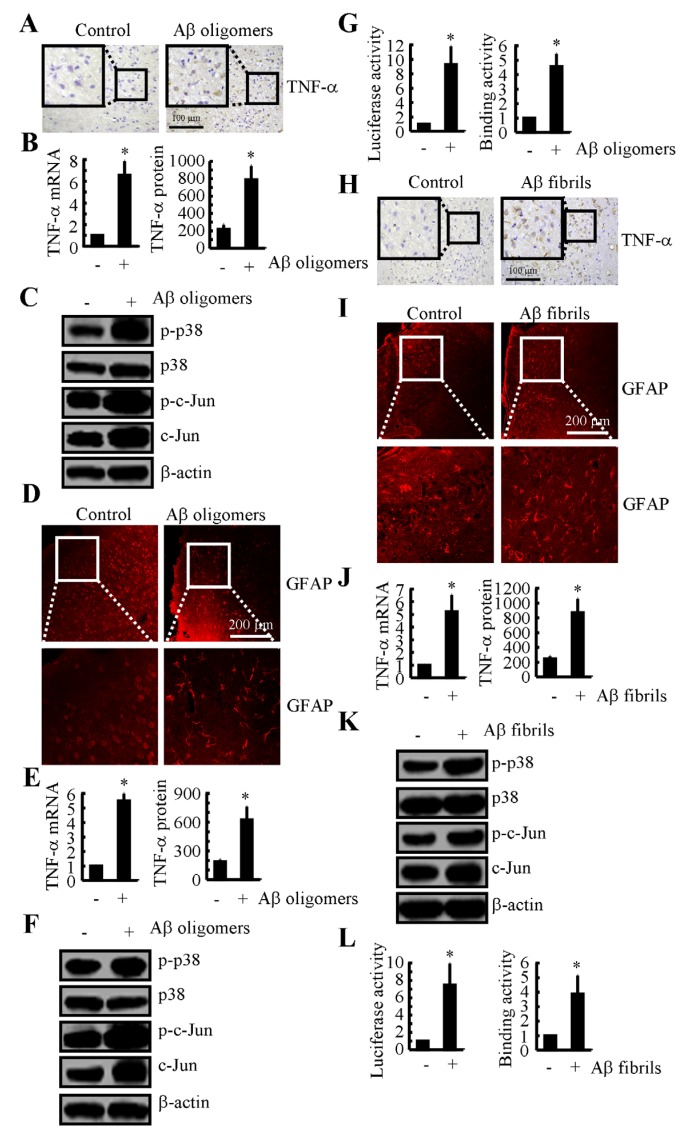
Both Aβ oligomers and fibrils have the ability to stimulate the expression of TNF-α by activating astrocyte *via* a p38- and c-Jun-dependent pathway **A.**-**D.** WT mice at the age of 3 months were injected with Aβ oligomers (1 µg/5 µl) for 24 h before the brains were harvested (*n* = 6). **A.** TNF-α immunoreactivity was determined *via* immunohistochemistry. **B.** TNF-α protein and mRNA levels were determined by TNF-α enzyme immunoassay kits and qRT-PCR, respectively. **C.** Levels of phosphorylated p38, total p38, phosphorylated c-Jun and total c-Jun were detected by immunoblotting using specific antibodies. Equal lane loading was demonstrated by the similarity of the intensity of the bands for total β-actin. **D.** The activity of astrocyte was determined by immunofluorescence. **E.**-**G.** D1A cells were treated with Aβ oligomers (1 µM) for 24 before the total mRNA and protein was extracted. **E.** TNF-α protein and mRNA levels were determined by TNF-α enzyme immunoassay kits and qRT-PCR, respectively. **F.** Levels of phosphorylated p38, total p38, phosphorylated c-Jun and total c-Jun were detected by immunoblotting using specific antibodies. Equal lane loading was demonstrated by the similarity of the intensity of the bands for total β-actin. **G.** The activity of the TNF-α promoter was determined by luciferase activity kits. The binding activity of AP-1 to the promoter of TNF-α was determined by ChIP assay. **H.**, **I.** Freshly sliced tissue was cultured with Aβ fibrils (1 µg/ml) for 24 h before immunostaining with TNF-a or GFAP antibody. **J.**-**L.** D1A cells were treated with Aβ fibrils for 24 h before total mRNA and protein was extracted. **J.** TNF-α protein and mRNA levels were determined by TNF-α enzyme immunoassay kits and qRT-PCR, respectively. **K.** Levels of phosphorylated p38, total p38, phosphorylated c-Jun and total c-Jun were detected by immunoblotting using specific antibodies. Equal lane loading was demonstrated by the similarity of the intensity of the bands for total β-actin. **L.** The activity of the TNF-α promoter was determined by luciferase activity kits. The binding activity of AP-1 to the promoter of TNF-α was determined by ChIP assay. *, *p < 0.05* with respect to the vehicle-treated control.

Even though PGI_2_-induced Aβ_1-42_ could not alleviate TNF-α suppression in the cells, these observations indicate that Aβ deposition might be critical for the TNF-α upregulation in the mice. To further validate this hypothesis, we injected Aβ oligomers (i.c.v.) into the ventricles of WT mice. The results demonstrated that TNF-α expression was upregulated (Figures 6A, 6B). In addition, the phosphorylation of p38 and c-Jun was upregulated in the Aβ_1-42_ oligomer-injected mice (Figure 6C). Consistent with the critical role of astrocyte in producing TNF-α, injection (i.c.v.) of Aβ_1-42_ oligomers into the ventricles of the WT mice stimulated the activity of GFAP (Figure 6D). In agreement with these *in vivo* observations, *in vitro* studies further verified the critical role of Aβ_1-42_ oligomers in stimulating the expression of TNF-α in D1A cells (Figures 6E-6G).

Because TNF-α is progressively upregulated during the course of AD development, we sought to understand the role of Aβ fibrils and APs in upregulating the expression of TNF-α. To further explore the role of the advanced aggregate forms of Aβ_1-42_ in TNF-α regulation, we sliced fresh brain specimens from WT mice (400 µm) for culturing. The results demonstrated that TNF-α was activated after 24 h of treatment with Aβ_1-42_ fibrils (Figure 6H). In addition, astrocyte activity was stimulated by the Aβ fibril treatment (Figure 6I). Similar results were obtained in D1A cells (Figure 6J). To further elucidate the underlying mechanism, we conducted experiments to determine the effects of Aβ fibrils on the activity of the p38 and c-Jun signalling pathways. The results demonstrated that the Aβ fibrils stimulated the activity of the TNF-α promoter by activating AP1 in D1A cells (Figures 6K, 6L). Therefore, our data revealed that not only Aβ_1-42_ oligomers but also Aβ_1-42_ fibrils have the ability to stimulate TNF-α expression by activating astrocyte, which produce high levels of TNF-α during the course of AD development.

### TNF-α overproduction accelerates the development of AD

**Figure 7:**
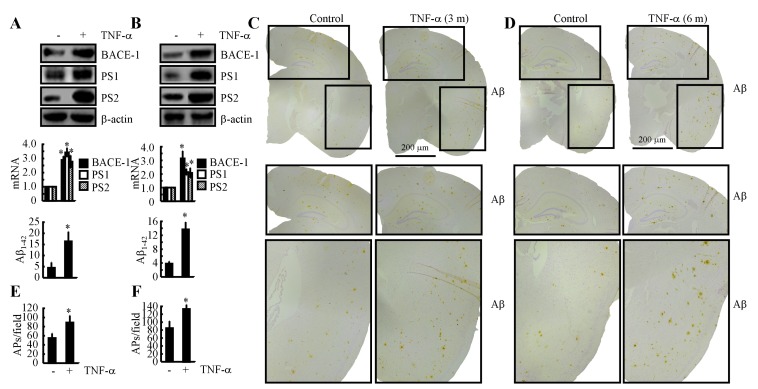
Intranasal administration of TNF-α accelerates Aβ deposition in APs by inducing the expression of BACE-1, PS1 and PS2 during the course of AD development **A.** TNF-α (10 ng/20 µl/d) was nasally administered to 3-month-old WT mice for 7 days (*n* = 10). The protein and mRNA expression of BACE-1, PS1 and PS2 were determined by western blot and qRT-PCR. The total amounts of β-actin and GAPDH served as internal controls. The production of Aβ_1-42_ was determined by western blot and Aβ_1-42_ enzyme immunoassay kits. **B.** n2a cells were treated with TNF-α (10 ng/ml) for 24 h before total mRNA and protein was extracted. The protein and mRNA expression of BACE-1, PS1 and PS2 were determined by western blot and qRT-PCR. The total amounts of β-actin and GAPDH served as internal controls. The production of Aβ_1-42_ was determined by western blot and Aβ_1-42_ enzyme immunoassay kits. **C.**-**F.** 3-month-old APP/PS1 mice were nasally administered TNF-α (10 ng/20 µl/d) for 3 or 6 months before Aβ deposition in APs was determined (*n* = 6). **C.**, **D.** Aβ immunoreactivity was determined using an immunohistochemistry assay. **E.**, **F.** APs/field in the cerebral cortex and hippocampus of APP/PS1 mice were analysed by counting the number of APs in images of the immunohistochemically stained tissue. *, *p < 0.05* with respect to the vehicle-treated control.

Because the mechanisms underlying the induction of TNF-α during the course of AD development in APP/PS1 mice had been elucidated, we decided to investigate the role of TNF-α in Aβ deposition. The results demonstrated that intranasally administering TNF-α (10 ng/20 µl/d) for 7 days clearly increased the expression of BACE-1, PS1 and PS2 and that these increases resulted in an acceleration of the production of Aβ_1-42_ (Figure 7A). This *in vivo* observation was further verified in n2a cells (Figure 7B). To further explore the role of TNF-α in Aβ aggregation, we further treated 3-month-old APP/PS1 mice for 3 months or 6 months. The results demonstrated that Aβ deposition in APs was clearly elevated after 3 or 6 months of treatment (Figures 7C-7F). These observations clearly demonstrated that TNF-α overproduction accelerated the production and aggregation of Aβ_1-42_ in APs, which exacerbate the development of AD.

When considered together, our data revealed that PGE_2_ stimulated the synthesis of TNF-α *via* an Aβ-dependent, AP-1 activation-mediated pathway in APP/PS1 Tg mice, an experimental AD model. Additionally, PGI_2_ attenuated the effects of PGE_2_ in stimulating the expression of TNF-α by decreasing the activity of the p38 and JNK/c-Jun pathways. Although PGI_2_ could upregulate the production of Aβ_1-42_, the induced Aβ_1-42_ could not reverse the inhibitory effects of PGI_2_ on TNF-α expression. In line with these *in vitro* and *in vivo* observations, TNF-α was further found to be responsible for accelerating the production and deposition of Aβ_1-42_. More importantly, both Aβ_1-42_ oligomers and Aβ_1-42_ fibrils had the ability to stimulate the expression of TNF-α, which could potentially aggravate the pathogenesis of AD by accelerating Aβ deposition in APs.

## DISCUSSION

Prior work has revealed the early induction of COX-2 and of its metabolic products during the course of AD development [1]. Therefore, we studied the role of COX-2 and its metabolic products in AD. As a powerful inducer of inflammation, COX-2 has been shown to induce the expression of TNF-α *via* its metabolic products [13, 14]. So, we investigated the role of PGE_2_ and PGI_2_ in regulating the expression of TNF-α during the course of AD development. Specifically, PGE_2_ stimulated the expression of TNF-α *via* an Aβ_1-42_-dependent, AP1-activating pathway. In contrast, PGI_2_ attenuated the effects of PGE_2_ on inducing the expression of TNF-α *via* a p38 and JNK/c-Jun-activating mechanism. Although Aβ_1-42_ reliably induced the expression of TNF-α by activating p38 and JNK/c-Jun, PGI_2_-induced Aβ_1-42_ might not be sufficient to reverse the inhibitory effects of PGI_2_. In agreement with these *in vitro* observations, we found that PGE_2_ and PGI_2_ antagonistically regulated the expression of TNF-α in an Aβ_1-42_-dependent manner. Moreover, both Aβ_1-42_ oligomers and Aβ fibrils had the ability to upregulate the expression of TNF-α, resulting in constitutively high levels of TNF-α during the course of AD development.

Accumulating evidence has demonstrated that PGI_2_ has the ability to suppress the expression of TNF-α. For instances, Vicil *et al.* [24] reported that TNF-α synthesis was inhibited by the PGI_2_ analogue beraprost sodium in lipopolysaccharide (LPS)-treated lung alveolar epithelial cells. In line with this observation, Yeh *et al.* [25] found that PGI_2_ analogues, including iloprost and treprostinil, suppressed LPS-induced TNF-α expression in human monocyte-derived dendritic cells. In addition, the inhibitory effects of PGI_2_ on TNF-α expression have also been confirmed in human monocytes [26], endothelial cells [27] and diabetic patients [28]. Along these lines, our data not only identified a suppressive role of PGI_2_ in the expression of TNF-α but also provided the first demonstration that PGI_2_ attenuated the effects of PGE_2_ in stimulating the expression of TNF-α. More importantly, the effects of PGI_2_ might be responsible for the cause for the failure of clinical trials for COX-2-specific inhibitors because these trials did not take into consideration the suppressive effects of PGI_2_ on the neuroinflammation of AD.

In view of the roles of PGE_2_ and PGI_2_ in regulating the production of Aβ_1-42_ [29], possible involvements of Aβ_1-42_ in mediating the regulatory effects of PGE_2_ and PGI_2_ on the expression of TNF-α are hypothesized. Although, to the best of our knowledge, there are no related reports concerning the role of PGE_2_ in upregulating the expression of TNF-α *via* Aβ_1-42_, the effect of PGE_2_ in increasing the ratio of Aβ_1-42_ and Aβ_1-40_ has been demonstrated in HEK293 cells, SH-SY5Y cells and APP23 mice [30]. In agreement with this result, EP4 overexpression in APP/PS1 Tg mice enhances the effect of PGE_2_ in increasing the ratio of Aβ_1-42_ and Aβ_1-40_ [10]. In addition, PGE_2_ treatment increases the production of Aβ_1-42_ in C57BL/6 mice [31]. More closely, mPGES-1 overexpression in Tg2576 mice accelerated the deposition of Aβ_1-42_ in APs [32]. For PGI_2_, it is also effective on stimulating the production of Aβ_1-42_ [29].

In line with the above observations, our data revealed that not only Aβ_1-42_ oligomers but also Aβ_1-42_ fibrils had ability to stimulate the expression TNF-α. In agreement with our data, Liu *et al.* [33] reported that Aβ_1-42_ has the ability to stimulate the synthesis of TNF-α in rat hippocampus. This observation was further confirmed by Lv *et al.* [34] , who suggested that the RAGE signalling pathway is important for TNF-α synthesis in Aβ_1-42_-treated BV-2 cells. In addition, peripheral administration of an anti-TNF-α receptor fusion protein counteracted the Aβ_25-35_-induced elevation of TNF-α and memory deficits in mice [35], suggesting that TNF-α acts downstream of Aβ to impair the learning ability of mice during the course of AD development. Because Aβ_25-35_ is the minimum unit of Aβ involved in the pathogenesis of AD, the injection of Aβ_25-35_ has been used to induce the expression of TNF-α in the astrocyte of the rat brain as an experimental model of AD [36, 37]. As expected, Aβ_1-42_ also induces the expression of TNF-α in AD experimental models [33]. However, these observations do not address the different aggregated forms of Aβ in the regulation of the synthesis of TNF-α. For example, Lv *et al.* [34] reported that Aβ_1-42_ oligomers have the ability to stimulate the expression of TNF-α in BV-2 cells. Like Aβ_1-42_ oligomers, fibrillar Aβ_1-42_ also has the ability to activate microglia, which potentially contribute to the pathogenesis of AD [38]. In addition, Veerhuis *et al.* [39] demonstrated that AP-associated proteins, including C1q and SAP, have the ability to enhance the effects of Aβ_1-42_ in inducing the expression of TNF-α in adult human microglia. Compared to Aβ_1-42_ fibrils, Aβ_1-42_ oligomers exerted a more potent effect on the expression of TNF-α in rat brain [40]. These phenomena were explained by Lindberg *et al.* [41] , who suggested that the structure of Aβ determines the nature of the cytokines that are released from rat microglia. Notably, this theory was further supported by White *et al.* [42], who suggested that the high levels of IL-1β that are induced by Aβ oligomers decrease over time, whereas Aβ fibrils induce high levels of IL-1β that are sustained over time. Along these lines, we could not exclude the possibility that Aβ oligomers have the ability to initiate the synthesis of TNF-α during the early stage of AD and that Aβ fibrils are responsible for sustaining high levels of TNF-α during the late stage of AD.

Although the above observations aquiescenced that microglia are the major source of TNF-α in primary cultured glials [43], they still could not negate the roles of astrocytes in producing TNF-α in primary cultured astrocytes [44]. Indeed, we extended the prior works to find that astrocytes are responsible for bridging the connections between Aβ and microglia in stimulating the expression of TNF-α. In line with our work [44], Choi *et al.* [45] recently reported that activated, but not non-activated-astrocytes are responsible for the synthesis of TNF-α, which acts as a molecular coordinator of neuron-glia communication. This observation was further supported by previous study showing that TNF-α expression was stimulated in lipopolysaccharide (LPS)-, interferon γ (IFNγ)- or IL-1β-stimulated astrocytes [46]. Lau *et al.* [47] also reported that TNF-α expression was also induced in traumatic and metabolic injury-activated brains. Since TNF-α was produced from both astrocytes and microglia in response to multiple stimulators, we further found that TNF-α is a molecular coordinator for mediating astrocytes and microglia communication. In another word, the microglia will produce TNF-α once astrocytes were activated and produced TNF-α. Reciprocally, astrocytes will produce TNF-α once microglia were activated and produced TNF-α. In addition, TNF-α was also identified to be expressed in primary cultured neurons. Even though TNF-α mediated the crosstalk between astrocytes and microglia, TNF-α neither exerts its biological function on astrocytes nor microglia. Interestingly, we found that TNF-α will translocate from astrocytes and microglia to neurons, which aggravate the neuroinflammation during the course of AD development and progression.

However, TNF-α is not passively involved in the development of AD. Consequently, our results demonstrated that TNF-α treatment accelerated the deposition of Aβ_1-42_ in APs by inducing the expression of BACE-1 and PS1/2 during the course of AD progression. Consistent with our findings, TNF-α was shown to have the ability to increase the expression of BACE-1 in APPsw Tg mice [20]. In addition, TNF-α stimulated the γ-cleavage of APP in HEK293 cells [18]. Indeed, TNF-α has the ability to induce the expression or phosphorylation of γ-secretases, including PS1, PS2 and NCT, in HEK293 and human neuronal cells [17, 19]. More directly, TNF-α plus IFNγ has been shown to induce the production of Aβ in human neuronal and extraneuronal cells [48]. A TNF-α inhibitor, 3,6’-dithiothalidomide, has been shown to block the effects of Aβ_1-42_ on the memory deficits of mice [49]. When considered together, these data clearly reveal the reciprocal roles of TNF-α on the induction of Aβ, which further aggravates AD.

Regarding the mechanism, we found that the p38 and AP-1 pathways are involved in mediating the antagonistic regulatory effects of PGE_2_ and PGI_2_ on the expression of TNF-α in D1A cells. In agreement with our observations, accumulating evidence indicates that the p38 and JNK/c-Jun pathways are involved in the regulation of the expression of TNF-α. However, although investigators tried to identify the mechanisms responsible for the suppression of the expression of TNF-α in astrocyte, this work did not extend to PGE_2_ and PGI_2_ [50-54]. This is not the first time we have demonstrated the involvement of the p38 and JNK/c-Jun pathways in regulating the expression of TNF-α; however, we extended these mechanisms to include the roles of PGE_2_ and PGI_2_ in regulating the synthesis of TNF-α. Nevertheless, we will not continue to discuss this mechanism. To keep the discussion focused, we though it was necessary to identify the cells that secreted TNF-α. According to our data, TNF-α seemed to be secreted from astrocyte, which then bind to neurons to exert their biological functions. In agreement with our findings, Frey *et al.* [55] reported that TNF-α was significantly induced in LPS-treated BV-2 cells. In line with this *in vitro* observation, our *in vivo* results further supported the notion that the deposition of Aβ activates astrocyte. Activated microglia elicit the expression of TNF-α, which influences the surrounding brain tissue [56]. More specifically, Clausen *et al.* [57] showed that CD11b^+^CD45^dim^ microglia are responsible for the synthesis of TNF-α. Because TNF-α was highly expressed in astrocyte, the question of why our TNF-α immunostaining results seemed to show expression in neurons could be easily raised. Our results demonstrated that TNF-α secreted from astrocyte will bind to the receptors of microglia and neurons to exert its biological functions. In line with our data, the results of Bhaskar *et al.* [58] also suggested that microglia-derived TNF-α drives AD-related neuronal cell cycle events. Taken together, these observations not only more clearly delineate the mechanism responsible for the upregulation of TNF-α during the course of AD development but also provide insight into the opposing roles of PGE_2_ and PGI_2_ in the learning ability of AD experimental model mice *via* their effects on TNF-α. More importantly, we identify a possible reason for the failure of clinical studies of COX-2-specific inhibitors by showing that the ratio of PGE_2_ to PGI_2_ is important for the pathogenesis of AD. If the COX-2-specific inhibitors could not return the imbalance between PGE_2_ and PGI_2_ to a physiological level, they could not produce a therapeutic effect against AD.

In conclusion, this study provides new evidence for the antagonistic roles of PGE_2_ and PGI_2_ in regulating the expression of TNF-α *in vitro* and *in vivo*. Specifically, we showed that PGE_2_ upregulates the expression of TNF-α *via* an Aβ-dependent, p38- and JNK/c-Jun-activating pathway. In contrast, we showed that PGI_2_ attenuates the effects of PGE_2_ in stimulating the expression of TNF-α. Because PGI_2_ only modestly induced Aβ_1-42_, the resulting Aβ_1-42_ induction was insufficient to alleviate the cells from PGI_2_-mediated TNF-α inhibition in an AP1-dependent manner. With regard to the critical role of Aβ_1-42_ in regulating the expression of TNF-α, we further discovered that Aβ_1-42_ oligomers and fibrils are responsible for the synthesis of TNF-α during the early and late stages of AD, respectively. Reciprocally, TNF-α binds to the receptors of neurons to aggravate AD by accelerating the deposition of Aβ_1-42_ in APs *via* the upregulation of the expression of BACE-1 and PS1/2. These findings provide new insight into the mechanisms of TNF-α regulation in the brain during the course of AD development.

## MATERIALS AND METHODS

### Reagents

PGI_2_, PGE_2_, Aβ_1-42_ and the inhibitors NS398, SB203580, and SP600125 were obtained from Sigma-Aldrich Corp (St. Louis, MO, USA). Antibodies against β-actin, p38, p-p38, c-Jun, p-c-Jun, COX-2, TNF-α, BACE-1, PS1, PS2, Iba1, GFAP, NeuN and Aβ were purchased from Cell Signaling Technology, Inc. (Danvers, MA, USA). The mouse TNF-α enzyme immunoassay kits were obtained from Raybiotech, Inc. (Norcross, GA, USA). The mouse Aβ_1-42_ enzyme immunoassay kits were obtained from Invitrogen (Carlsbad, CA, USA). Dual luciferase reporter assay kits were purchased from Promega (Beijing, P. R. China). The chromatin immunoprecipitation (ChIP) EZ-ChIP kit was purchased from Merck Millipore (Beijing, P. R. China). All reagents for the qRT-PCR and SDS-PAGE experiments were purchased from Bio-Rad Laboratories. All other reagents were from Invitrogen (Carlsbad, CA, USA) unless otherwise specified.

### Cell culture

Mouse astrocyte D1A or microglia BV-2 cells were grown (37 °C and 5% CO_2_) on 6-cm tissue culture dishes (10^6^ cells per dish) in appropriate medium. In a select set of experiments, the cells were grown in serum-free medium for an additional 24 h before incubation with inhibitors in the absence or presence of PGI_2_ or PGE_2_, as previously described [59]. In separate experiments, D1A cells were cocultured with neuroblastoma 2a (n2a) cells or the cultured slices of mouse brains before determining the expression of TNF-α by immunostaining. In brief, D1A cells were seeded in the upper chamber of transwell and n2a or cultured slices were cultured in the lower chamber of transwell. After 24 h, the n2a or slices were immunostained with TNF-α.

### Transgenic mice

The wild type (WT), APP/PS1 transgenic mice [B6C3-Tg (APPswe, PSEN1dE9) 85Dbo/J (Stock Number: 004462)] or COX-2 transgenic mice (Stock Number 010703) were obtained from The Jackson laboratory (Bar Harbor, ME, USA). Genotyping was performed at 3-4 weeks after birth. The mice were housed in a controlled environment under a standard room temperature, relative humidity and 12-h light/dark cycle with free access to food and water. Mice were randomly separated into several groups and each group contains 10 mice for treatment. The general health and body weights of animals were monitored every day. The brains of animals from the different groups were collected under anesthesia and perfusion as previously described [60, 61].

### Intracerebroventricular injection (i.c.v)

NS398, PGE_2_, PGI_2_, Aβ, or Aβ antibody or vehicle (PBS) solutions were injected (i.c.v) into WT or APP/PS1 transgenic mice as previously described [60, 61]. In selected experiments, the WT mice were injected (i.c.v) with the CSF of APP/PS1 mice. Briefly, stereotaxic injections were placed at the following coordinates from the bregma: mediolateral: -1.0 mm; anteroposterior: -0.22 mm; and dorsoventral: -2.8 mm. Following injections, each mouse recovered spontaneously on a heated pad. The reliability of injection sites was validated by injecting trypan blue dye (Invitrogen) into separate cohorts of mice and observing staining in the cerebral ventricles. Twenty-four hours after injection, mice were harvested under anesthesia and perfusion as previously described [60, 61].

### Organotypic slice culture of brain tissue

Brain tissues were freshly collected from WT C57BL/6 mice at 6 months of age. Serial sections (400-µm thick) were cut using a chopper without fixation. The tissue sections were immediately cultured in DMEM/high glucose medium with 10% fetal bovine serum (FBS). In a separate set of experiments, the tissues were grown in serum-free medium for an additional 24 h before incubation with Aβ oligomers or fibrils, as previously described [60, 61]. The tissue sections were fixed and immunostained with TNF-α antibody by an immunohistochemical staining kit.

### Luciferase assays and live animal imaging

The D1A cells that were transfected with an TNF-α promoter were pre-seeded in one side of a ventricle. PGI_2_, PGE_2_ or vehicle (PBS) solutions were then injected (i.c.v) into the other side of ventricle. At different time intervals, mice were anesthetized and injected (i.c.v) with luciferin into the cerebral ventricle, which was preseeded with D1A cells. The scan was performed exactly after 5 min of luciferin introduction as previously described [60, 61]. All images were taken and analyzed using Bruker *in vivo* imaging systems (MS FX PRO, Carestream, U.S.A).

### Luciferase promoter constructs

A 2, 000-base pair (bp) TNF-α promoter construct, corresponding to the sequence from -1774 to +226 relative to the transcription start site of the 5’-flanking region of human TNF-α gene, was generated from human genomic DNA, using specifically designed forward 5’-TATCGAT AGGTACCGAGCTCCACTTCAGCCCCAGCAGTGT-3’ and reverse 5’ GAT CGCAGATCTCGAGTTTTGGGGGAGTGCCTCTTC-3’ primers incorporating *Sac 1* and *Xho1* restriction sites (underlined) at the 5’ ends and 3’ends, respectively. The amplicon was then inserted upstream of the luciferase reporter gene in the pGL3-basic vector (Promega). All construct sequences were confirmed using DNA sequencing.

### Promoter assay

Firefly and *Renilla* luciferase activities were measured by use of the Dual-Luciferase Report Assay kit (Promega). Firefly luciferase activities were normalized to the *Renilla* luciferase controls. Data are expressed as ratios of shear to static normalized firefly luciferase activity unless otherwise stated.

### ChIP assay

This assay was performed using the EZ ChIP kit following the manufacturer’s instructions (Upstate Biotechnology) as described previously [62-65]. Forward (F) and reverse (R) primers for TNF-α promoter amplification by qPCR are as follows: F-atgcacccagctttcagaag and R- tctcggtttcttctccatcg.

### Quantitative real-time PCR (qRT-PCR)

qRT-PCR assays were performed with the MiniOpticon Real-Time PCR detection system (Bio-Rad) using total RNA and the GoTaq one-step Real-Time PCR kit with SYBR green (Promega) and the appropriate primers as previously described [66]. The GenBank accession number and forward and reverse primers for mouse GAPDH and BACE-1 are provided in our previous publications [59, 67, 68]: mouse TNF-α (NM_013693) F- tagctcccagaaaagcaagc, R- gggaacttctcatccctttg; PS1 (NM_008943) F- gcttgtaggcgcctttagtg, R- catctgggcattctggaagt; PS2 (NM_011183) F- aagaacgggcagctcatcta, R- tccagacagccaggaagagt. The gene expression values were normalized to those of GAPDH.

### Western blot analysis

Tissues or cells were lysed in radio-immune precipitation assay buffer (25 mM Tris-HCl [pH 7.6], 150 mM NaCl, 1% NP-40, 1% sodium deoxycholate, and 0.1% SDS) that contained a protease inhibitor cocktail (Pierce Chemical Company). The protein content of the cell lysates was determined using the bicinchoninic acid (BCA) protein assay reagent (Pierce Chemical Company). The total protein lysates (4 μg) were separated using SDS-PAGE, transferred to a membrane, and probed with a panel of specific antibodies. Each membrane was only probed with one antibody. β-actin was used as a loading control. All western hybridizations were performed at least in triplicate using a different cell preparation each time.

### Transfection

The coding sequence of p38 was generated from mouse total mRNA, using specifically designed forward 5’- TTAAACT TAAGCTTGGTACCATGTCGCAGGAGAGGCCCAC-3’ and reverse 5’-GATATCTGCA GAATTCTCAGGACTCCATTTCTTCTTGGTCAAG-3’ primers, which was incorporated to pCDNA 3.1 (+) vectors by Kpn 1 and EcoR 1 restriction sites (underlined) at the 5’ ends and 3’ ends, respectively. Similarly, the coding sequence of c-Jun was inserted to pCDNA 3.1 (+) vectors by Kpn 1 and EcoR 1 restriction sites using specifically designed forward 5’-TTAAACTTAAGC TTGGTACCATGACTGCAAAGATGGAAACGACC-3’ and reverse 5’-GATATC TGCAGAATTCTCAAAACGTTTGCAACTGCTGC-3’ primers. Cells were transfected with 1.6 µg/6 cm dish of p38 or c-Jun cDNA plasmids. In control experiments, the cells were transfected with 100 nM scrambled siRNA. The transfected cells were allowed to recover for at least 12 h in growth medium and then incubated overnight in serum-free medium before extraction. In live animal scanning experiments, the D1A cells were transfected with 1.6 µg/6 cm dish TNF-α promoter plasmid for 48 h before screening the positive clones by puromycin. The positive clones were then injected or preseeded in the ventricles of mice.

### Aβ_1-42_ preparation

The methods for preparing Aβ oligomers or fibrils had been described previously [69]. In brief, freeze-drying Aβ_1-42_ protein (Stock Number: A9810, Sigma, St. Louis, MO, USA) was initially monomerized by dissolving it to a final concentration of 1 µg/µl in 100% hexafluoroisopropanal (HFIP) and the solution was aliquoted in sterile eppendorf tubes. HFIP was then evaporated under vacuum and the peptide was stored at -20 °C before reconstituent. For preparing Aβ_1-42_ oligomers, the peptide was initially resuspended in dimethylsulfoxide (DMSO) to 20 µg/µl with water bath ultrasonication for 10 min and the solution was then diluted to a final concentration of 0.2 mg/ml in phenol red-free F-12 media, and incubated at 4°C for 24 h. To prepare Aβ_1-42_ fibrils, Aβ_1-42_ was resuspended in sterile Milli Q water and incubated at 37°C for 1 week before use.

### Immunohistochemistry

Brain tissues were collected from WT or APP/PS1 transgenic mice. In selected experiments, brain tissues were collected after injection (i.c.v) of PGI_2_ (2 µg/5 µl) or PGE_2_ (2 µg/5 µl). Serial sections (5-µm thick) were cut using a paraffin microtome (Leica, RM2235, Germany). Sections were first rehydrated in a graded series of ethanol and submerged in 3% hydrogen peroxide to eliminate endogenous peroxidase activity. The activity of TNF-α or Aβ was determined by immunostaining TNF-α or Aβ antibody using an immunohistochemical staining kit, following the manufacturer’s instructions (Invitrogen, Carlsbad, CA, USA).

### Immunofluorescence

Brain tissues were collected from WT or APP/PS1 transgenic mice. In selected experiments, brain tissues were collected after injection (i.c.v) of PGI_2_ (2 µg/5 µl) or PGE_2_ (2 µg/5 µl). Serial sections (10-µm thick) were cut using a cryostat (Leica, CM1850, Germany). Slides were stained with GFAPor Iba1 antibody with Alexa Fluor 555 secondary antibodies (Cell Signaling Technology, Inc., Danvers, MA, USA) before observing under confocal microscopy (Leica, TCS-SP8, Leica).

### Measurement of the TNF-α or Aβ_1-42_ concentration in the culture medium or the brain of mice

The TNF-α or Aβ_1-42_ levels in the media of both control and pharmacologically treated cells or the brain of mice were determined using TNF-α or Aβ_1-42_ enzyme immunoassay kits following the manufacturer’s instructions. The total protein used for ELISA was used as a loading control, and the results are expressed as pg of TNF-α or pmol Aβ_1-42_ per mg of total protein.

### Human brain samples

Human brain samples were obtained from New York Brain Bank, serial numbers TT4263 (early stage of AD, the patient is 73-years-old man who was diagnosed as a mild AD patient), T4308 (middle stage of AD, the patient is 86-years-old man who was diagnosed as moderate AD patient), T4339 and T4304 (late stage of AD, the patients are 88-years-old woman and 84 years-old woman who were diagnosed as severe and end stage of AD patients).

### Animal committee

All animals were handled according to the care and use of medical laboratory animals (Ministry of Health, Peoples Republic of China, 1998) and all experimental protocols were approved by the Laboratory Ethics Committees of College of Life and Health Sciences of Northeastern University.

### Statistical analysis

All data are represented as the mean ± S.E. of at least three independent experiments. The statistical significance of the differences between the means was determined either using Student’s *t*-test [59].

## SUPPLEMENTARY MATERIALS FIGURE


