# Supplementary material for: Prostaglandin I2 is responsible for ameliorating prostaglandin E2 stress in stimulating the expression of tumor necrosis factor α in a β-amyloid protein -dependent mechanism
Source: Oncotarget. 2017 Jun 13;8(61):102801–19. doi: 10.18632/oncotarget.18462 (PMC5732691; doi:10.18632/oncotarget.18462)
Supplement: Supplementary file 1 [file oncotarget-08-102801-s001.pdf]

Prostaglandin I_2 is responsible for ameliorating prostaglandin E_2 stress in stimulating the expression of tumor necrosis factor α in a β-amyloid protein -dependent mechanism

SUPPLEMENTARY MATERIALS

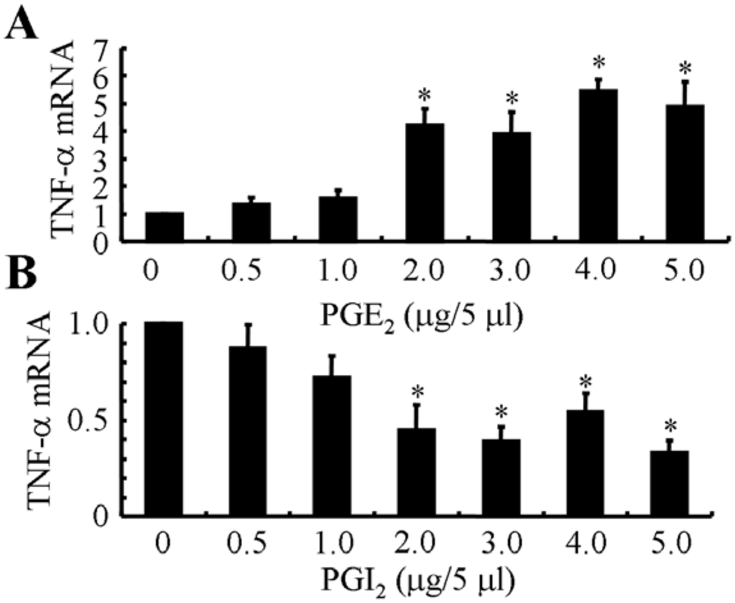

Supplementary Figure 1: Involvement of PGE_2 and PGI_2 in opposite regulating the expression of TNF-α in C57BL/6 mice. The C57BL/6 mice was injected (i.c.v) with PGE_2 or PGI_2 for 24 h before the brains were harvested. TNF-α mRNA level was determined by qRT-PCR. *, $p<0.05$ with respect to the vehicle-treated control.
